# A Systematic Review of the Evidence on the Effectiveness and Cost-Effectiveness of Mass Screen-and-Treat Interventions for Malaria Control

**DOI:** 10.4269/ajtmh.21-0325

**Published:** 2021-09-07

**Authors:** Sooyoung Kim, Verah Nafula Luande, Joacim Rocklöv, Jane M. Carlton, Yesim Tozan

**Affiliations:** ^1^ School of Global Public Health, New York University, New York, New York;; ^2^ Department of Public Health and Clinical Medicine, Umeå University, Umeå, Sweden;; ^3^ Center for Genomics and Systems Biology, Department of Biology, New York University, New York, New York

## Abstract

Malaria elimination and eradication efforts have stalled globally. Further, asymptomatic infections as silent transmission reservoirs are considered a major challenge to malaria elimination efforts. There is increased interest in a mass screen-and-treat (MSAT) strategy as an alternative to mass drug administration to reduce malaria burden and transmission in endemic settings. This study systematically synthesized the existing evidence on MSAT, from both epidemiological and economic perspectives. Searches were conducted on six databases (PubMed, EMBASE, CINALH, Web of Science, Global Health, and Google Scholar) between October and December 2020. Only experimental and quasi-experimental studies assessing the effectiveness and/or cost-effectiveness of MSAT in reducing malaria prevalence or incidence were included. Of the 2,424 citation hits, 14 studies based on 11 intervention trials were eligible. Eight trials were conducted in sub-Saharan Africa and three trials in Asia. While five trials targeted the community as a whole, pregnant women were targeted in five trials, and school children in one trial. Transmission setting, frequency, and timing of MSAT rounds, and measured outcomes varied across studies. The pooled effect size of MSAT in reducing malaria incidence and prevalence was marginal and statistically nonsignificant. Only one study conducted an economic evaluation of the intervention and found it to be cost-effective when compared with the standard of care of no MSAT. We concluded that the evidence for implementing MSAT as part of a routine malaria control program is growing but limited. More research is necessary on its short- and longer-term impacts on clinical malaria and malaria transmission and its economic value.

## INTRODUCTION

Malaria is a vector-borne disease that affected over 200 million people in 2019,^1^ and imposes a significant economic burden on endemic countries. According to the 2019 WHO malaria report, 19 countries collectively account for 85% of global malaria burden.^1^ All of these high-burden countries are also resource poor, and (except for India) are located in sub-Saharan Africa (SSA). In these countries, *Plasmodium falciparum* and *P. vivax* infections account for the majority of malaria cases.^1^ In the past two decades, great strides have been made in malaria control, and this has increased enthusiasm toward malaria elimination with the ultimate goal of its eradication.^2^ Worryingly, progress on malaria elimination and eradication has stalled globally in the last few years,^1^ and it has been argued that we have reached the limits of what we can achieve with the imperfect tools and limited resources we have.^3^ Currently, a serious obstacle to malaria control and elimination efforts is asymptomatic infections that provide *Anopheles* mosquitoes with a silent parasite reservoir that sustain transmission in endemic areas.^4^

Mass drug administration (MDA) is being reexamined by the malaria community as an intervention strategy as it remains one of the few strategies whose full potential has yet to be realized in endemic areas.^3^ In MDA, irrespective of the presence of symptoms or infection, every member of a population living in a defined geographic area receives a full therapeutic course of an effective antimalarial drug.^5^ Typically, MDA is repeated at intervals, and each round is conducted over a short time span. MDA not only clears symptomatic infections, but also has the potential to reduce the prevalence of asymptomatic parasitemia that is chronic and often goes undetected and untreated in clinical settings.

Currently, MDA is not recommended as a core malaria intervention.^3^ This is because long-term use of MDA in areas with stable malaria transmission has raised a number of concerns, including that frequent administration of antimalarial drugs in a population may present safety issues.^6^^–^^10^ Perhaps a greater concern is the possibility of inducing drug resistance as a result of repeated use of in the context of MDA.^6^ Hence, the WHO limits the use of MDA as part of a multipronged approach to reduce transmission for achieving malaria elimination in low-to-moderate transmission settings where there is access to case management and other malaria control interventions, or in complex emergency and epidemic settings where healthcare systems are overwhelmed and incapable of providing routine malaria services.^5^

A mass screen-and-treat (MSAT) strategy has been proposed as an alternative to MDA to achieve malaria elimination in endemic settings. The principle undergirding MSAT is active detection of infections, both symptomatic and asymptomatic, in a given population, using malaria diagnostic tools such as rapid diagnostic tests (RDTs), light microscopy (LM), and molecular methods such as polymerase chain reaction (PCR), prior to treatment with antimalarials. MSAT may hold comparative advantage to MDA in minimizing the excess use of antimalarial drugs on those who do not need them, thus reducing the risk of antimalarial drug resistance and enabling better use of resources. The 2015 WHO recommendation on MSAT was stricter compared with MDA, limiting its use to only complex emergencies and epidemics, and was informed by the findings of only a handful of existing studies. This systematic review built on this initial assessment and reviewed all experimental and quasi-experimental studies that assessed the effectiveness of MSAT in reducing malaria prevalence or incidence since then. In particular, we synthesized the existing evidence on MSAT, from both epidemiological and economic perspectives, to identify knowledge gaps and provide guidance on future research and implementation of MSAT in the context of malaria control and elimination programs.

## MATERIALS AND METHODS

### Protocol and registration.

This systematic literature review followed the Preferred Reporting Items for Systematic Reviews and Meta-Analyses protocol (Supplemental Appendix 1). The protocol of the systematic review was registered in International Prospective Register of Systematic Reviews (PROSPERO; CRD42020214610).^7^

### Search strategy and selection criteria.

Searches were conducted on six different databases, namely, PubMed, EMBASE, CINALH, Web of Science, Global Health, and Google Scholar, between October and December 2020. The search strategy is built using the MeSH terms for malaria and other search terms. All databases, expect EMBASE, were searched using these MeSH terms, with all the subheadings, as well as other key words. For EMBASE, similarly, Emtree terms associated with malaria and other search terms were used, with all the subcategories. The search strategy details for each database are given in Supplemental Appendix 1. The intervention of interest for this review was defined as detecting and treating human malaria infections using a mass testing and treatment approach. Given the heterogeneity of the terminology used in the malaria literature, we included both “screen-and-treat” and “test-and-treat” as search terms, as well as their variants. We limited our inclusion criteria to intervention trials that were conducted one-time or at regular intervals but over a short period of time, using RDTs, LM, or PCR. Only studies reporting epidemiological and/or cost-effectiveness outcomes were included. The outcomes of interest included effect size estimates of the intervention in reducing malaria prevalence or incidence, expressed as risk difference (RD), risk ratio (RR), incidence rate ratio (IRR), hazard ratio (HR), or odds ratio (OR), as well as cost-effectiveness estimates, expressed as incremental cost-effectiveness ratio (ICER). To ensure the quality of evidence on intervention effectiveness, studies only with experimental or quasi-experimental designs were considered. The review focused on published peer-reviewed literature between January 1, 2000 and November 1, 2020, given the wide expansion in use of RDTs in the field in early 2000s, following the WHO’s consultation on the topic in 1999, which was published in 2000.^8^^,^^9^ Searches were performed without any language restrictions.

We excluded studies with observational designs with no control group and model-based studies of intervention effectiveness, as well as study protocols, literature reviews, and conference abstracts. We also excluded any study that did not include all components of the intervention strategy. For example, any mass treatment intervention without a testing component, a focal test-and-treat (FTAT) intervention targeting only a narrow group of people around index cases, or any community-based treatment intervention based on passive surveillance of cases in clinical settings were excluded from the review.

### Study selection.

After removing duplicates, two reviewers (SK and VL) independently screened all studies by title and abstract based on the inclusion and exclusion criteria. Any disagreements between the two reviewers were resolved by consensus. Full texts of selected studies were then reviewed by the same two reviewers to confirm inclusion. We further reviewed the references of all included studies to identify other potentially eligible studies. Any disagreements were resolved by a third reviewer (YT), who reviewed and validated the final list of included studies. Excluded studies were recorded with reasons for exclusion.

### Data collection and management.

To minimize errors and bias, two reviewers (SK and VL) independently extracted data from included studies using a data extraction form that was developed and tested for reliability. Information extracted included country of study, year of publication, study period, study setting regarding malaria transmission (i.e., seasonality, transmission intensity, and predominant parasite species), study design, sample characteristics (i.e., sample size, age, gender), statistical and economic outcome measures reported, and intervention characteristics (target population, duration and frequency of MSAT rounds, malaria diagnostic test, and antimalarial drug used). The supplemental materials of included studies were also reviewed to identify any relevant information. The data extracted by the two reviewers were first compared and then merged into one database after resolving any disagreements. The data extraction form and all extracted data are available in Supplemental Appendix 1 and 2.

### Synthesis of results.

Extracted data was cleaned by the principal reviewer (SK) for further analysis. Extracted data on intervention effects were categorized by effect size calculation method and yielded five mutually exclusive categories: RD, RR, HR, IRR, and OR. Economic outcomes included ICER and other reported cost estimates associated with the intervention, such as cost per tested or treated. Outcome data were also broadly grouped into four categories for the purposes of narrative synthesis and meta-analysis: incidence, prevalence, costs, and cost-effectiveness.

### Meta-analysis.

The selection of outcomes and the statistical methods for meta-analysis were guided by Harrer et al.^10^ Based on the data we extracted, outcome measures that were reported consistently across multiple individual studies were selected for meta-analysis—namely, IRR and HR for incidence, and OR and RR for prevalence. We combined estimates of IRR and HR to calculate a pooled estimate of the incidence of malaria, whereas for malaria prevalence, two separate meta-analyses were conducted using ORs and RRs.^11^ When multiple measures were reported for an outcome in a study, the reviewers established a set of criteria for the prioritization and selection of outcomes. Specifically, in efficacy trials, we prioritized the results of per-protocol (PP) analysis over intent-to-treat (ITT) analysis when both analyses were conducted. For prevalence and incidence, we prioritized outcomes by malaria diagnostic tool in descending order from PCR, LM, RDT to clinical diagnosis to best capture the effectiveness of the intervention on asymptomatic/subclinical malaria cases. Lastly, we considered results over all cases of malaria rather than species-specific results.

When multiple (≥ 2) individual studies targeting the same population reported on the same outcome measures, the results were pooled in R software (version 3.6.3) using the package “meta” for random-effect meta-analysis.^12^ Forest plots reporting individual study outcomes and pooled effect sizes were generated together with I^2^ values and 95% confidence intervals to assess heterogeneity in the included studies. Furthermore, outcomes reported for specific vulnerable populations (e.g., school children, pregnant women) were assessed separately in a sub-analysis, and a pooled effect size was derived, when data are appropriate for meta-analysis.

### Risk of bias in individual studies.

Two individual reviewers (SK, VL) conducted independent risk of bias assessment on all included studies using the Cochrane Collaboration’s tool.^11^ The assessed domains using this tool included random sequence generation, allocation concealment, masking of participants and personnel, masking of outcome assessment, incomplete outcome data, selective reporting, and other biases. Since many studies followed cluster-randomized design where masking of individual participants was not possible because of ethical reasons, the assessment on this criterion not only considered the existence of participant masking in the study design, but also the extent to which masking or the lack of masking of participants are likely to affect the results to be biased. The assessment was done at the study level and any disagreements between the reviewers were reconciled by consensus. The summarized risk of bias was used to inform our synthesis of findings and discussion.

## RESULTS

### Summary of selected literatures.

**Figure 1.**
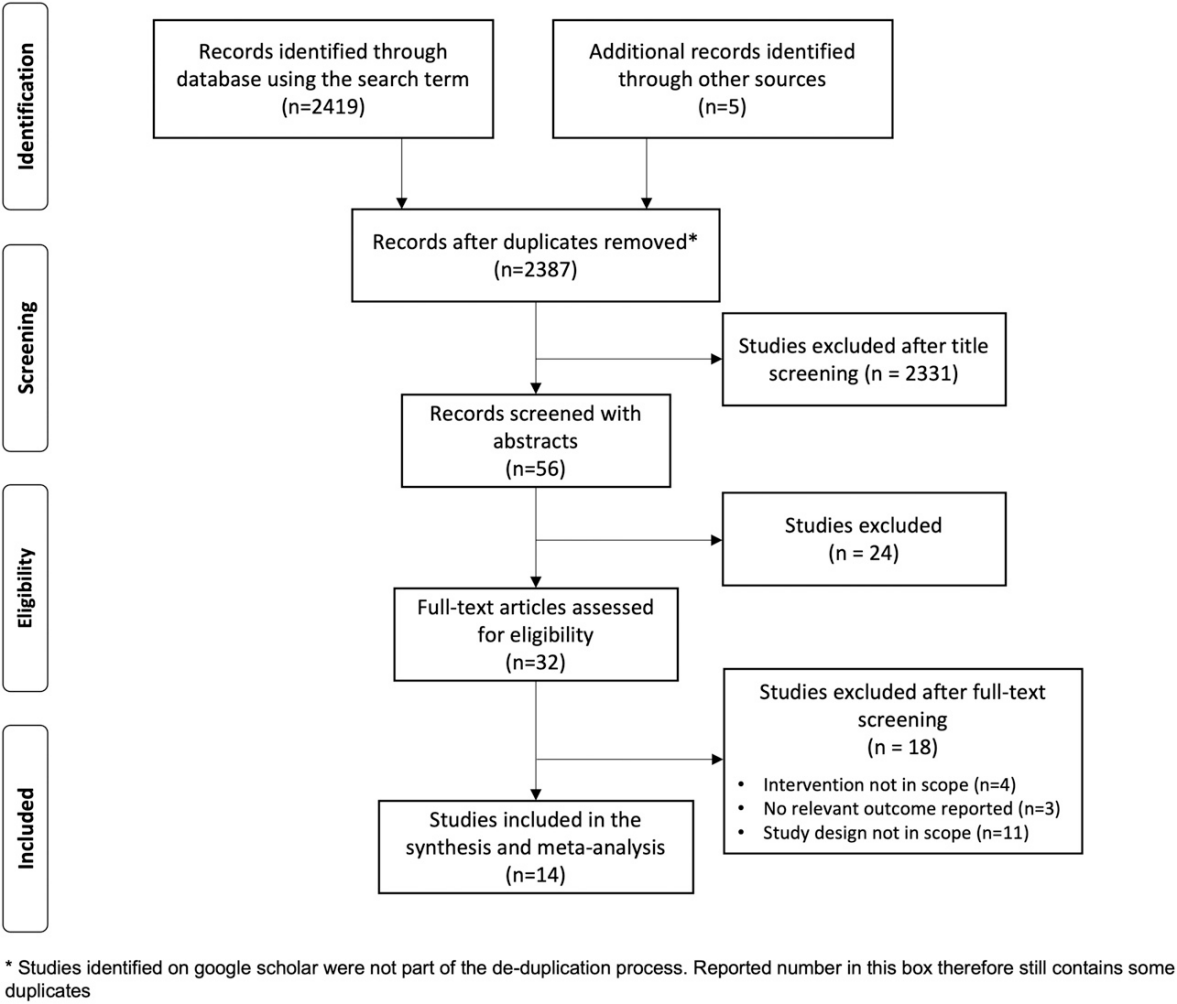
Preferred Reporting Items for Systematic Reviews and Meta-Analyses (PRISMA) flow diagram of the search strategy.

The database search identified a total of 2,424 studies. After removing duplicates, 2,387 studies were screened by title and abstract. Of these, 2,355 did not meet the inclusion criteria. We reviewed 32 full-text studies, of which 14 were deemed eligible for inclusion (Figure 1). These 14 studies presented data from 11 different intervention trials (hereafter referred to as “trials”). Table 1 provides a summary of the key features of each study.

**Table 1:** Summary characteristics of the 14 included studies in the systematic literature review

Study	Country	Study design	Transmission intensity	Target population	Sample size	% children under 5	% children under 18	Study duration and frequency	Intervention coverage	Test used	Treatment used	Control group	Measured outcomes	Target parasite*
Tagbor et al., 2010	Ghana	Non-masked RCT	Perennial with peak	Pregnant women and infants (outcome only)	3,333	0.00%	9.81%	Three rounds of intervention over 20 weeks, and additional 16 weeks of follow-up	Not mentioned	RDT	SP, AQ+AS	SP-IPTp at gestational week 24, 32, 36	anemia, new-borne outcome, prevalence, maternal outcome	U
Tiono et al. (a), 2013,	Burkina Faso	Non-masked Cluster RCT	Seasonal with peak (Jun–Nov)	General population	14,075	16.33%	47.89%	Four rounds of intervention over 12 months	96.10%	RDT	AL	no treatment of asymptomatic carriers	prevalence, others, parasite density	*PF*
Tiono et al. (b), 2013,	Burkina Faso	Non-masked Cluster RCT	Seasonal with peak (Jun–Nov)	General population	14,075	16.33%	47.89%	4 rounds of intervention over 12 months	96.10%	RDT	AL	no treatment. LLIN was distributed before the intervention and the usage was checked every 2 months.	incidence, anemia, prevalence	*PF*
Halliday et al., 2014	Kenya	Non-masked Cluster RCT	Moderate and perennial with two peaks (Apr–Jul, Sep–Nov).	School children	5,176	0.00%	100.00%	Five rounds of intervention over 20 months, with additional 6 months follow-up	66.8% (84% for > 4 rounds)	RDT	AL	No MSAT	anemia, prevalence	PF
Larsen et al., 2015	Zambia	Non-masked Cluster RCT	Moderate with peak (Mar–May)	Pregnant women and infants (outcome only)	811	100.00%	100.00%	Three rounds of intervention over 6 months, with additional 6 months of follow-up	88%	RDT	AL	Delayed MTAT after the intervention	prevalence, incidence	U
Silumbe et al., 2015	Zambia	Non-masked Cluster RCT	Moderate with peak (Mar–May)	General population	135,649	Not reported	Not reported	Three rounds of intervention over 6 months	66.2% based on the program administration data (88.3% according to the census data)	RDT	AL	Delayed MTAT after the intervention	cost	U
Natama et al., 2018	Burkina Faso	Non-masked Cluster RCT	Perennial with peak (Jul–Nov)	Pregnant women and infants (outcome only)	761	–	–	Three rounds of intervention over 24 weeks (from first ANC visit to delivery), with additional 1 year of follow-up	88%	RDT	AL	receive IPTp-SP (intermittent preventive treatment)	incidence, parasite density, others, prevalence	PF
Sutanto et al., 2018	Indonesia	Non-masked Cluster RCT	Low with peak (Aug–Sep)	General population	1,295	Not reported	26.56%	Three rounds of intervention over 3 months, with additional 3 months of follow-up	> 90% (treatment adherence)	Microscopy	DHAP	MST0: no MSAT intervention,	prevalence, others, incidence	*PF and PV*
												MST2: received 2 rounds with 10 weeks-interval between Jun–August OR No MSAT		
Ahmed et al., 2019	Indonesia	Non-masked Cluster RCT	Sumba: low transmission	Pregnant women and infants (outcome only)	1,598	–	–	Three rounds of intervention over 34 weeks, with additional 8 weeks of follow-up	Median three follow-ups (range 1–6) with 3.1-month interval (IQR 2.1–4.0)	RDT	DHAP	Single test-and-treat at the first antenatal visit OR monthly administration of treatment regimen without screening (IPT)	maternal outcome, new-borne outcome	U
			Papua: moderate perennial transmission											
Cosmic Consortium, 2019	multicountry	Non-masked Cluster RCT	Gambia/Burkina Faso: high and seasonal (Jul–Dec)	Pregnant women and infants (outcome only)	4,731	–	–	Four rounds of intervention over 24 weeks (from first ANC visit to delivery)	Average of 3–4 visits per woman	RDT	AL	Two rounds of IPTp-SP during their second to third trimester, only tested and treated when symptomatic	others, maternal outcome, prevalence, anemia, new borne outcome	U
			Benin: moderate and perennial with 2 peaks (Apr–Jul, Oct–Nov)											
Kuepfer et al., 2019	India	Non-masked Cluster RCT	Varying with peak (Jun–Oct)	Pregnant women and infants (outcome only)	6,868	–	5.04%	Three rounds of intervention over 22 weeks, with additional 26 weeks of follow-up	28% (54% for two visits)	RDT	SP	test-and-treat only when symptomatic during the ANC visit	maternal outcome, new borne outcome	unspecified
Conner et al., 2020	Senegal	Non-masked, nonrandomized cluster CT	Low with peak (Jul–Jan)	General population	22,170	19.41%	56.19%	One round of intervention and 6-month follow-up	77%	RDT	AL+DHAP	control 1: case investigation only	incidence, cost	unspecified
												control 2: weekly fever screen, test and treat (PECADOM++)		
Desai et al., 2020	Kenya	Non-masked Cluster RCT	Perennial, high with two peaks (May–Jul, Nov–Dec)	General population	1,066	10.51%	44.75%	Six rounds of intervention over 7 months	75–96% (measured each round)	RDT + PCR, RDT + microscopy	DHAP	Standard of care	incidence	unspecified
Samuels et al., 2020	Kenya	Non-masked Cluster RCT	Perennial, high with two peaks (May–Jul, Nov–Dec)	General population	2,012	13.97%	40.76%	Six rounds of intervention over 19 months with additional 5 months of follow up	75.0–77.5% during year 1 rounds, 81.9–94.3% in year 2.	RDT + PCR	DHAP	Standard of care	prevalence	unspecified

PF = *P. falciparum*; PV = *P. vivax*; U = Unspecified.

Of the 11 trials, three took place in Asian countries while the rest were conducted in SSA. All trials, except one, had a cluster-randomized design (*N* = 10), and none was masked to participants due to feasibility and ethical considerations. The intervention was targeted at pregnant women in five trials, and at school children in one trial. The target population in the remaining five trials was the community as a whole.

Malaria transmission intensity ranged from low to high across study sites. Five trials explicitly mentioned perennial transmission, while all study sites reported seasonal variability in transmission, with either one peak (*N* = 9) or two peaks (*N* = 2). Some trials involved multiple study sites with differing malaria transmission intensities. Overall, three trials included study sites in low transmission settings;^13^^–^^15^ five trials had at least one study site with moderate transmission;^15^^–^^19^ and two trials included study sites in high transmission settings.^19^^–^^22^ Three trials did not specify transmission intensity.^23^^–^^26^

In studies targeting the community as a whole, the number of MSAT rounds ranged between one and six over a period of 3–12 months. In all studies, control arms received standard care for malaria, while two studies had an additional study arm to assess the effectiveness of: 1) different rounds of MSAT,^13^ or 2) weekly fever screen, test, and treat.^14^ Post-intervention assessment of epidemiological outcomes was carried out either immediately after the final round of MSAT or up to 6 months.

In studies targeting pregnant women, MSAT was initiated in the first trimester of pregnancy and continued up until time of delivery. While some studies aimed to test and treat pregnant women for malaria monthly, three to four MSAT rounds were typically conducted. In only one study, women and their babies were followed up to 12 months after birth for longer-term outcome assessment. The single study targeting school children conducted five MSAT rounds over 20 months, and children were followed up to 6 months post-intervention.

### Evidence of effectiveness in improving malaria outcomes.

**Figure 2.**
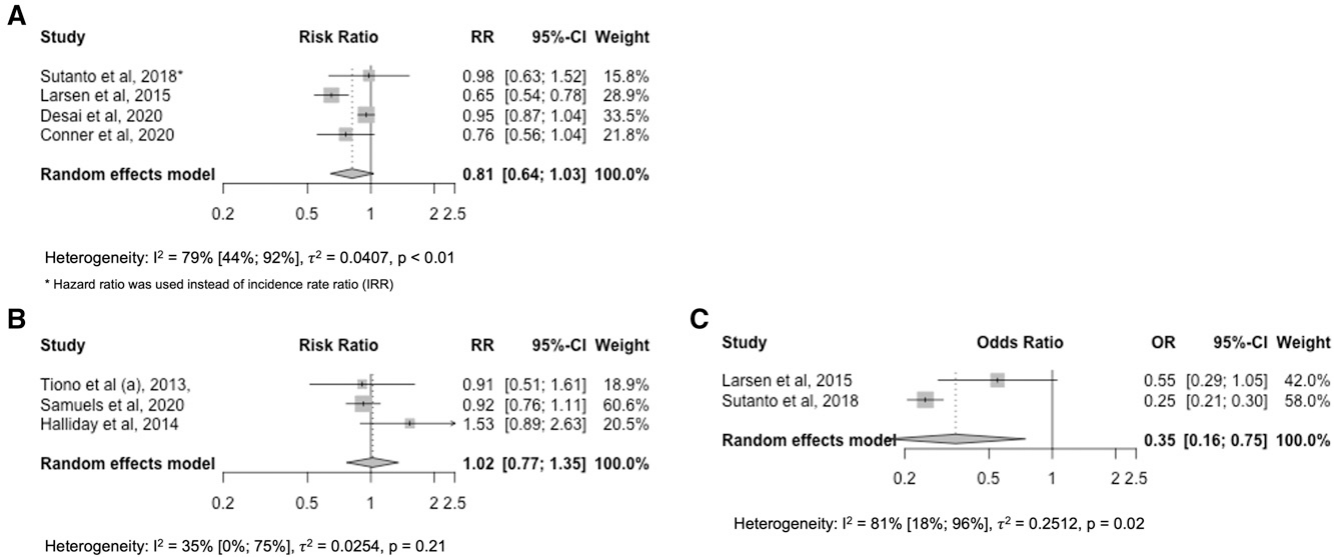
Pooled estimates of epidemiological outcomes from the 14 included studies using random-effect meta-analysis methods.

The incidence of malaria was reported as IRR in three studies,^14^^,^^17^^,^^20^ as HR in two studies,^13^^,^^20^ and as RR in one study.^13^ The pooled effect size for the incidence of malaria, combining IRRs and HRs, was 0.81 (95% CI: 0.64–1.03, I^2^ = 79% [95% CI: 44%; 92%]), suggesting a marginal and statistically nonsignificant decrease in incidence (Figure 2A). The prevalence of malaria was reported as OR in two studies^13^^,^^17^ and as RR in three studies.^16^^,^^21^^,^^24^ The pooled effect size for the prevalence of malaria was significant for OR (0.35, 95% CI: 0.16–0.75, I^2^ = 81% [95% CI: 18%; 96%]), but not for RR (1.02, 95% CI: 0.77–1.35, I^2^ = 35% [95% CI: 0%; 75%]) (Figure 2B and C). However, two out of three meta-analyses showed high inter-study heterogeneity, as expressed in I^2^ value close to 100%, posing challenges in interpreting the pooled effects. All data used in these meta-analyses are provided in Supplemental Appendix 1.

### Evidence of cost-effectiveness.

Two studies reported the costs of MSAT intervention,^14^^,^^18^ whereas only one of these studies assessed its cost-effectiveness.^18^ The cost-effectiveness analysis was conducted from a provider perspective, and estimated an ICER of US$894 per DALY averted for a three-round MSAT intervention in Zambia. Conducted between June and November 2012, the intervention was estimated to prevent over 16,000 cases and 30 deaths from malaria in a target population of 135,649 across 18 catchment areas and to avert over 1,300 DALYs. As the mean estimated ICER was lower than Zambia’s gross domestic product per capita of US$1,414, the intervention was found to be cost-effective compared with no MSAT.^27^

In Zambia, the cost per test administered was estimated at US$4.39 and ranged between US$3.45–US$5.94 depending on catchment area size and number of MSAT rounds.^18^ A study conducted in Senegal estimated the cost per person tested at US$14.3 for one round of MSAT covering 22,170 people over 6 months,^14^ which was found to be significantly higher than the cost of other malaria control interventions in this setting. In both studies, cost estimates were most sensitive to training and transportation costs. These findings suggest that once MSAT becomes part of a routine malaria program, there may be economies of scale with significant reductions in implementation costs.

### Effect of the intervention on specific vulnerable populations.

Interventions targeting pregnant women were not effective in reducing the prevalence of maternal malaria infection measured at both placental (pooled OR: 1.05, 95% CI: 0.85–1.31) and peripheral (pooled OR: 0.94, 95% CI: 0.76–1.15) levels. The pooled estimates for each outcome are summarized in Supplemental Appendix 1.

### Risk of bias assessment.

**Figure 3.**
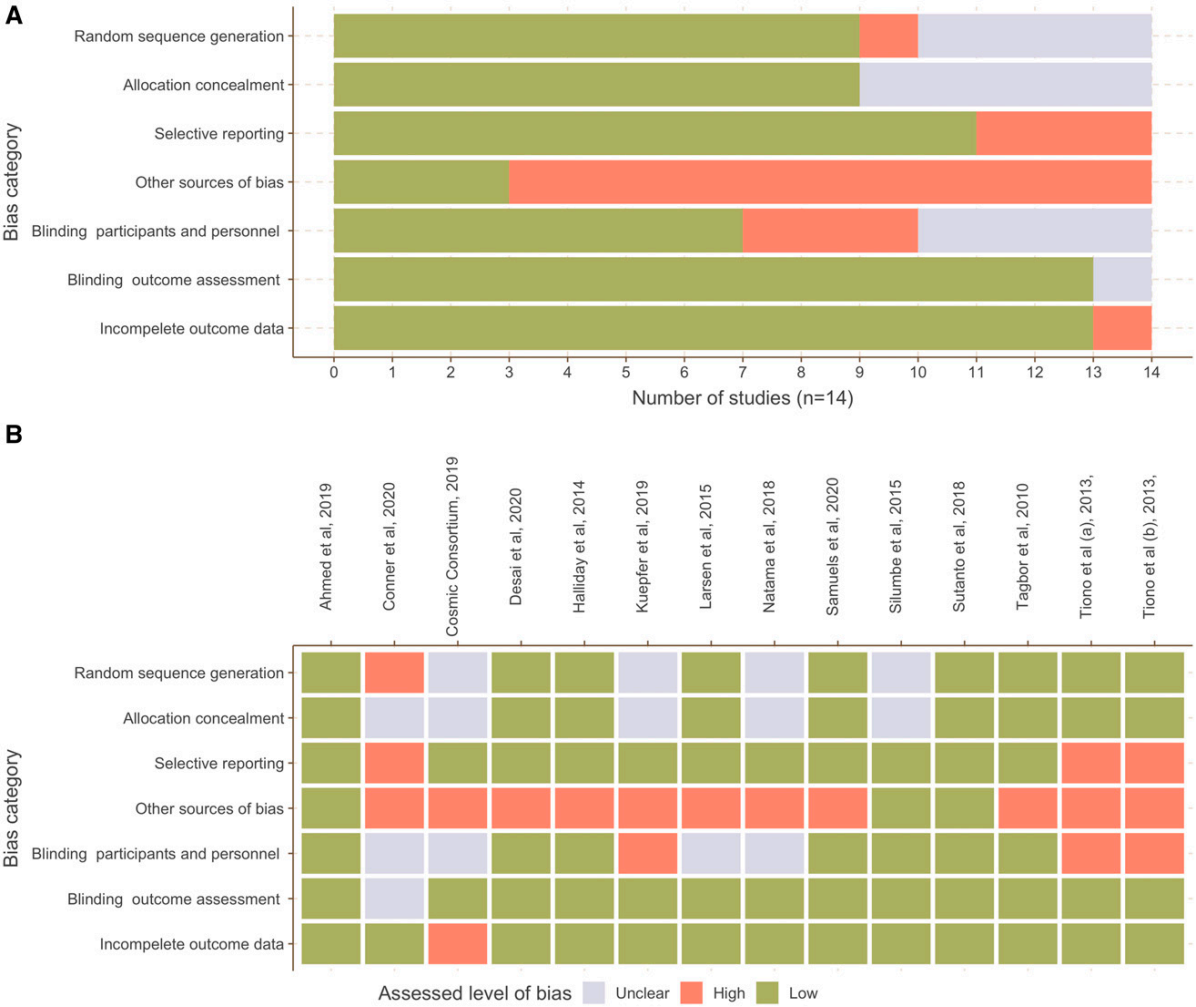
Risk of bias assessment for the 14 studies included in the systematic literature review. This figure appears in color at www.ajtmh.org.

The risk of bias was found to be generally low across all assessed domains but one (i.e., other sources of bias), and the quality of information provided in studies to assess risk was, overall, high (Figure 3). None of the studies was masked to participants, and most measured outcomes were not likely to be affected by this lack of masking. Three studies^24^^–^^26^ were, however, assessed to have high risk of bias due to non-masked study design; two studies^24^^,^^25^ reporting on the same trial relied on self-reported number of healthcare visits to measure incidence post-intervention; and one study^26^ reported significantly lower adherence rate among participants in the intervention arm compared with the control arm. Another study among pregnant women^19^ was evaluated to have a high risk of bias because women in the intervention arm were more frequently tested, and this potentially resulted in a higher estimate of malaria incidence in the intervention arm compared with the control arm. Another potential source of bias in outcome measurements was mitigated in 13 of the 14 studies by masking laboratory staff to the allocated study arm. Only one study did not provide sufficient information to assess this bias.

Regarding the measurement of epidemiological outcomes, a high risk of bias was observed in 11 of the 14 studies. The prevalence of malaria was most commonly assessed by RDTs. Only five studies^13^^,^^15^^,^^24^^–^^26^ used PCR. In four of the five studies, the target population was pregnant women, and placental malaria was the principal outcome of interest. As a result, the evaluation of MSAT’s impact in reducing malaria prevalence was limited by the detection of cases by conventional RDTs or LM. Hence, the potential impact of MSAT on the prevalence of asymptomatic parasitemia remains poorly quantified.

Similar biases affected the measurement of malaria incidence in the trials. Out of the nine studies that included incidence as an outcome measure, six used patient records from health facilities to retrospectively measure this outcome post-intervention. This led to an exclusion of symptomatic cases that did not seek care, asymptomatic cases that could have been detected by RDT or LM, and sub-patent cases that could only be detected by PCR. Given that in most malaria endemic areas, the majority of cases are asymptomatic,^4^^,^^28^ this could have influenced the results of these trials.

## DISCUSSION

To the best of our knowledge, this is the first systematic literature review on the effectiveness and the cost-effectiveness of MSAT, and the second attempt to revisit this intervention since the WHO Malaria Policy Advisory Committee (MPAC) issued a recommendation on its use in 2015.^5^ In the absence of sufficient evidence, the WHO MPAC discouraged the use of MSAT in situations other than complex emergencies and epidemics.^29^ This recommendation was informed by a review of only four studies that evaluated MSAT-like interventions, including focal screening-and-test (FSAT)—which is similar to, but different from MSAT, for instance, in terms of geographic scope. Two of these four studies were included in this systematic review^17^^,^^25^ while the other two did not meet our eligibility criteria and hence were excluded.^30^^,^^31^ Cook et al.^30^ conducted the MSAT intervention in Zanzibar, Tanzania, but only reported a before–after comparison of the malaria prevalence in the target population. Stresman et al.^31^ conducted and reported the results of sentinel-based focal screening to explore an alternative strategy to MDA or MSAT, but did not report any outcome related to the effectiveness of treatment of individuals identified through screening. However, both studies raised the potential advantage of introducing screening before treatment in comparison to mass treatment approach, such as MDA.^30^^,^^31^

Our review includes a total of 12 additional studies with an experimental design that were not reviewed by the MPAC^16^^,^^18^^,^^23^^,^^24^ and also published after 2015.^13^^–^^15^^,^^19^^–^^22^^,^^26^ The results of our meta-analysis reaffirm that the effects of MSAT on malaria incidence and prevalence are marginal and statistically nonsignificant, and there are currently few studies available for evidence synthesis. It is important to note that a total of 11 trials were eligible to be included in the final synthesis, and meta-analyses were conducted with as few as two to three studies using average intervention effects given the limited number of trials. Further, the trials included study sites with varied transmission intensities, and also reported on a variety of outcome measures. Hence, we were unable to conduct a meta-analysis by transmission setting. In addition, most of the studies reported a mix of significant and nonsignificant intervention effects, with the majority being nonsignificant, which made interpretation of the results difficult. However, a handful of trials conducted in low^13^^,^^14^ and moderate^17^ malaria transmission settings suggested the potential impact of mass test-and-treat (MTAT) in reducing malaria incidence and prevalence, when measured by microscopy or PCR. On the other hand, the trials that were set in high malaria transmission settings^20^^,^^21^ showed mixed effects in reducing clinical cases.

This systematic review demonstrates that the existing epidemiological evidence on the effectiveness of MSAT is still limited and highlights deficiencies in trial design and reporting that warrant further attention. First, incidence and prevalence estimates were largely based on symptomatic and patent infections due to the low sensitivity of the diagnostic methods used. Almost all studies used RDTs and/or microscopy to detect malaria cases pre-, during, and post-intervention; however, it is well-established that the sensitivity of these technologies is suboptimal compared with PCR.^32^ Even the most sensitive RDTs are estimated to have less than 80% sensitivity compared with PCR.^33^ The reliability of RDTs is even lower for non-*falciparum* species when coinfections with other pathogens precede or exist due to the antigen–antibody cross-reactions.^4^ The choice of diagnostic technology also presents a major challenge in real-life implementation settings, and this highlights the importance of improving the insufficient sensitivity of existing field diagnostics to detect asymptomatic infections. Only four studies assessed the effectiveness of MSAT in reducing the incidence of asymptomatic or sub-patent infections,^15^^,^^22^^,^^24^^,^^25^ Two of which targeted pregnant women and their newborns.^15^^,^^22^ Tiono et al.^24^^,^^25^ showed a temporary reduction in the prevalence of asymptomatic carriers during the second and third round of MSAT; however, the reduction became nonsignificant after the implementation of all four rounds. There was no significant reduction in the incidence of asymptomatic infection among newborns during the 12-month follow-up period after birth.^22^ Ahmed et al.^15^ compared sub-patent–level malaria infections across different malaria transmission settings, but was not able to deduce the effectiveness of MSAT in reducing the incidence or prevalence sub-patent malaria infections.

Second, while most studies included in this review provided a detailed description of the intervention protocol, we failed to systematically extract information on intervention coverage and level of adherence to treatment across all included studies and were not able to assess the role of these factors on intervention efficacy. Thus, the observed differences in intervention effectiveness across included studies can potentially stem from higher adherence to intervention protocols. The WHO recommendation emphasized the importance of high intervention coverage for MDA, MSAT, and FTAT strategies.^5^ Even if the majority of included studies reported relatively high coverage, ranging between 74% and 96%, the studies did not provide information on adherence levels to antimalarial treatment. Some studies conducted both ITT and PP analyses,^15^^,^^17^^,^^23^ while most of the studies provided results based on only one method and did not justify their choice of method.

Third, intervention duration and follow-up periods were relatively short, which did not allow for an assessment of the longer-term effects of MSAT. With the exception of 2 studies, where the intervention was delivered over 20 months and the follow-up period was 4–6 months,^16^^,^^21^ all studies were conducted within a 12-month period. In sum, the limited evidence makes it challenging to understand whether these unsatisfactory results are due to poor sensitivity of the diagnostic methods used, insufficient coverage of the target populations, inadequate adherence to antimalarial treatment, treatment failure due to antimalarial resistance, insufficient duration of intervention, or a combination of these factors.

Existing mathematical modeling studies sheds light on some of the pending questions. Griffin et al.^34^ studied the effectiveness of MSAT in six different hypothetical African settings where long-lasting insecticidal nets (LLINs) were widely distributed (> 80%), and assumed that RDTs had a high diagnostic sensitivity similar to microscopy along with full treatment adherence among those who tested positive, as well as an intervention duration of 25 years. This model-based study showed that the combination of MSAT with scaled-up LLIN usage was effective to reduce parasite prevalence to less than 1% in low-to-moderate transmission settings.^34^ Another modeling study using the findings of a cohort study conducted in the Peruvian Amazon where the prevalence of asymptomatic malaria, mostly sub-patent, was estimated at 5–14%, showed that three consecutive rounds of MSAT with the start of the dry season would result in significant reductions in malaria incidence and prevalence, but at least 5 consecutive years of intervention would be required to eliminate the disease in the area.^35^ Based on the findings of these model-based studies, it is plausible that the trials included in this review failed to address the dynamics asymptomatic and sub-patent malaria infections, an area that is yet to be better understood.^28^

We also reviewed the existing evidence on the cost-effectiveness of MSAT, an important consideration for policymakers and program managers to assess the feasibility and sustainability of this intervention in resource-constrained settings. Cost per test administered was higher than other nontreatment interventions, but the one study that calculated ICER concluded that the intervention was cost-effective compared with no MSAT.^18^ The potential for further reduction in intervention costs was also suggested if implementation continued over years at scale since the initial training of staff would require a large investment. When compared with the ICER of MDA from a study conducted in the same country,^36^ it seemed even plausible that MSAT would be more cost-effective than MDA.

This review has a number of limitations. First, despite our best efforts to finetune the search strategy by including all relevant search terms related to MSAT, it is important to note the heterogeneity of nomenclature, which might have led to the exclusion of potentially eligible studies. Of the 14 studies included in this review, only two shared the same nomenclature for the intervention (community-wide screening and treatment)^24^^,^^25^ since these studies were based on the same trial. In the abbreviated form, there was more consistency: MTAT was used by five studies;^14^^,^^17^^,^^18^^,^^20^^,^^21^ community-based scheduled screening and treatment was used by two;^19^^,^^22^ and two studies used Intermittent Screen and Treat.^15^^,^^16^ None of the studies used MSAT, which is the terminology used by WHO.^5^^,^^29^ In addition, two studies that we encountered during the literature search but did not meet our eligibility criteria, described the intervention as an example of FSAT,^31^^,^^35^ although the description of the intervention strategy was closer to MSAT than FSAT. We believe that this review serves as a good starting point for streamlining the nomenclature in order for the intervention to be more systematically documented and reviewed in the future. Toward this end, we consider that both MSAT and MTAT explicate the key characteristics of the intervention around mass screening and treating well.

Second, as mentioned previously, the various outcome measures reported across studies made it challenging to obtain pooled estimates of intervention effects. This heterogeneity across studies also precluded an investigation of intervention effects by malaria transmission setting. Moving forward, to undertake a more robust assessment of the effectiveness of MSAT, there is a need for guidelines on the key outcomes to be measured in intervention trials.

In conclusion, the evidence for implementing MSAT as part of a routine malaria control program has grown since the WHO MPAC in 2015, but is limited. The time-limited nature of implementation and the simplicity of MSAT hold certain advantages to implement this intervention in hard-to-reach and resource-poor settings, and humanitarian crises and emergencies, where healthcare systems are disrupted. Compared with MDA, MSAT is likely to have more significant longer-term benefits in terms of reducing the development of antimalarial resistance and operational costs. However, due to the complex dynamics of malaria transmission and the myriad of factors that may affect program implementation, further research is needed to bolster current evidence on the effectiveness and cost-effectiveness of MSAT across different malaria transmission settings.

## Supplemental Material


Supplemental materials
