# Supplementary material for: A Systematic Review of the Evidence on the Effectiveness and Cost-Effectiveness of Mass Screen-and-Treat Interventions for Malaria Control
Source: Am J Trop Med Hyg. 2021 Sep 7;105(6):1722–31. doi: 10.4269/ajtmh.21-0325 (PMC8641306; doi:10.4269/ajtmh.21-0325)
Supplement: Supplementary file 1 [file tpmd210325.SD1.pdf]

Supplemental Appendix 1

A systematic review of the evidence on the effectiveness and cost-effectiveness of mass screen-and-treat interventions for malaria control

Sooyoung Kim[1], Verah Nafula Luande[2], Joacim Rocklöv[2], Jane M. Carlton[1,3], Yesim Tozan[1*]

1 School of Global Public Health, New York University, New York, NY, USA

2 Department of Public Health and Clinical Medicine, Umeå University, Umeå, Sweden

3 Center for Genomics and Systems Biology, Department of Biology, New York University, New York, NY, USA.

1. PRISMA Check-list

Section/topic	#	Checklist item	Reported on page #
**TITLE**			
Title	1	Identify the report as a systematic review, meta-analysis, or both.	Reported on the title (p1)
**ABSTRACT**			
Structured summary	2	Provide a structured summary including, as applicable: background; objectives; data sources; study eligibility criteria, participants, and interventions; study appraisal and synthesis methods; results; limitations; conclusions and implications of key findings; systematic review registration number.	Reported in the abstract (p2)
**INTRODUCTION**			
Rationale	3	Describe the rationale for the review in the context of what is already known.	pp5-6
Objectives	4	Provide an explicit statement of questions being addressed with reference to participants, interventions, comparisons, outcomes, and study design (PICOS).	p6 (line 105 – 114)
**METHODS**			
Protocol and registration	5	Indicate if a review protocol exists, if and where it can be accessed (e.g., Web address), and, if available, provide registration information including registration number.	p6 (line 117-121)
Eligibility criteria	6	Specify study characteristics (e.g., PICOS, length of follow-up) and report characteristics (e.g., years considered, language, publication status) used as criteria for eligibility, giving rationale.	pp6-7 (line 132-160)
Information sources	7	Describe all information sources (e.g., databases with dates of coverage, contact with study authors to identify additional studies) in the search and date last searched.	p6 (line 123-130)
Search	8	Present full electronic search strategy for at least one database, including any limits used, such that it could be repeated.	Supplementary material 2
Study selection	9	State the process for selecting studies (i.e., screening, eligibility, included in systematic review, and, if applicable, included in the meta-analysis).	p8 (line 162-170)
Data collection process	10	Describe method of data extraction from reports (e.g., piloted forms, independently, in duplicate) and any processes for obtaining and confirming data from investigators.	pp8-9 (line172-191)
Data items	11	List and define all variables for which data were sought (e.g., PICOS, funding sources) and any assumptions and simplifications made.	pp8-9 (line172-191) & Supplementary material 3
Risk of bias in individual studies	12	Describe methods used for assessing risk of bias of individual studies (including specification of whether this was done at the study or outcome level), and how this information is to be used in any data synthesis.	p12 (line 232-237)
Summary measures	13	State the principal summary measures (e.g., risk ratio, difference in means).	pp11-12 (line

			206-230)
Synthesis of results	14	Describe the methods of handling data and combining results of studies, if done, including measures of consistency (e.g., I^2) for each meta-analysis.	pp10-11 (line 193-204)
Risk of bias across studies	15	Specify any assessment of risk of bias that may affect the cumulative evidence (e.g., publication bias, selective reporting within studies).	p12 (line 232-237)
Additional analyses	16	Describe methods of additional analyses (e.g., sensitivity or subgroup analyses, meta-regression), if done, indicating which were pre-specified.	p12 (line 227-230)
**RESULTS**			
Study selection	17	Give numbers of studies screened, assessed for eligibility, and included in the review, with reasons for exclusions at each stage, ideally with a flow diagram.	Figure 1
Study characteristics	18	For each study, present characteristics for which data were extracted (e.g., study size, PICOS, follow-up period) and provide the citations.	pp12-14 (line241-272) & Table 1
Risk of bias within studies	19	Present data on risk of bias of each study and, if available, any outcome level assessment (see item 12).	pp17-18 (line 337-372)
Results of individual studies	20	For all outcomes considered (benefits or harms), present, for each study: (a) simple summary data for each intervention group (b) effect estimates and confidence intervals, ideally with a forest plot.	Supplementary material 3 and 4
Synthesis of results	21	Present results of each meta-analysis done, including confidence intervals and measures of consistency.	pp14-16 (line 274-325) & Supplementary material 4
Risk of bias across studies	22	Present results of any assessment of risk of bias across studies (see Item 15).	pp17-18 (line 337-372)
Additional analysis	23	Give results of additional analyses, if done (e.g., sensitivity or subgroup analyses, meta-regression [see Item 16]).	pp16-17 (line 327-335) & Supplementary material 5
**DISCUSSION**			
Summary of evidence	24	Summarize the main findings including the strength of evidence for each main outcome; consider their relevance to key groups (e.g., healthcare providers, users, and policy makers).	pp 18-20 (line 375-413)
Limitations	25	Discuss limitations at study and outcome level (e.g., risk of bias), and at review-level (e.g., incomplete retrieval of identified research, reporting bias).	pp 20-25 (line 414-522)
Conclusions	26	Provide a general interpretation of the results in the context of other evidence, and implications for future research.	pp 25-26 (line 523-533)
**FUNDING**			

Funding	27	Describe sources of funding for the systematic review and other support (e.g., supply of data); role of funders for the systematic review.	p1 (line7-9)

From: Moher D, Liberati A, Tetzlaff J, Altman DG, The PRISMA Group (2009). Preferred Reporting Items for Systematic Reviews and Meta-Analyses: The PRISMA Statement. PLoS Med 6(7): e1000097. doi:10.1371/journal.pmed1000097

For more information, visit: **www.prisma-statement.org**.

2. Search Terms

Database	Search terms	# of articles identified
PubMed / MEDLINE	malaria<br><br>AND<br><br>(“mass test*” OR “mass screen*” OR “focal test*” OR “focal screen*” OR “MSAT” OR “MTAT” OR “FSAT” OR “FTAT” OR “active case finding” OR “active case detection” OR “test and treat” OR “screen and treat” OR “test-and-treat” OR “screen-and-treat” OR “testing and treatment” OR “screening and treatment”)<br><br>AND<br><br>(season OR seasonal OR annual OR quarterly OR community OR rural)<br><br>AND<br><br>(effect* OR impact OR “odds ratio” OR “relative risk” OR “risk reduction” OR prevalence OR incidence OR efficacy OR evaluation OR epidemiolog* OR “cost-effectiveness” OR “economic evaluation” OR “economic analysis” OR “cost effectiveness”)<br><br>AND<br><br>(“developing countries” OR “developing country” OR “medically underserved area” OR “medically underserved areas” OR LMIC OR “low income countries” OR “low income country” OR “middle income countries” OR “middle income country” OR “resource poor” OR “low resource” OR “third world country” OR “third world countries” OR “less developed country” OR “less developed countries” OR “least developed country” OR “least developed countries” OR Africa OR “Central Asia” OR “Western Asia” OR “Southeastern Asia” OR “Indian Ocean Islands” OR “Central America” OR “South America” OR “Eastern Europe” OR Transcaucasia OR “Caribbean Region” OR “Pacific Islands” OR Afghan OR Afghani OR Afghanistan OR Bangladesh OR Bangladeshi OR Benin OR Beninese OR “Burkina Faso” OR Burkinabe OR Burundi OR Burundian OR Cambodia OR Cambodian OR “Central African Republic” OR “Central African” OR Chad OR Chadian	259

	OR Comoros OR Comoran OR Congo OR Congolese OR Eritrea OR Eritrean OR Ethiopia OR Ethiopian OR Gambia OR Gambian OR Guinea OR Guinean OR Haiti OR Haitian OR Kenya OR Kenyan OR Korea OR Korean OR Kyrgyz OR Kyrgyzstan OR Liberia OR Liberian OR Madagascar OR Malagasy OR Malawi OR Malawian OR Mali OR Malian OR Mozambique OR Mozambican OR Myanmar OR Myanmarese OR Burmese OR Nepal OR Nepalese OR Niger OR Nigerian OR Rwanda OR Rwandan OR “Sierra Leone” OR “Sierra Leonean” OR Somalia OR Somalian OR Tajikistan OR Tajik OR Tadzhik OR Tanzania OR Tanzanian OR Togo OR Togolese OR Uganda OR Ugandan OR Zimbabwe OR Zimbabwean OR Angola OR Angolan OR Armenia OR Armenian OR Belize OR Belizean OR Bhutan OR Bhutanese OR Bolivia OR Bolivian OR Cameroon OR Cameroonian OR “Cape Verde” OR “Cape Verdean” OR “Cape Verdean” OR “Cote d'Ivoire” OR “Ivory Coast” OR Djibouti OR Egypt OR Egyptian OR “El Salvador” OR Salvadoran OR Fiji OR Fijian OR Georgia OR Georgian OR Ghana OR Ghanaian OR Guatemala OR Guatemalan OR Guyana OR Guyanese OR Honduras OR Honduran OR Indonesia OR Indonesian OR India OR Indian OR Iraq OR Iraqi OR Kiribati OR Kosovo OR Kosovar OR Laos OR Lao OR Laotian OR Lesotho OR “Marshall Islands” OR Marshallese OR Mauritania OR Mauritanian OR Micronesia OR Micronesian OR Moldova OR Moldovan OR Mongolia OR Mongolian OR Morocco OR Moroccan OR Nicaragua OR Nicaraguan OR Nigeria OR Nigerian OR Pakistan OR Pakistani OR Papua New Guinea OR Papua New Guinean OR Paraguay OR Paraguayan OR Philippines OR Filipino OR Samoa OR Samoan OR “Sao Tome” OR Santomean OR Senegal OR Senegalese OR “Solomon Islands” OR “Solomon Islander” OR “Sri Lanka” OR “Sri Lankan” OR Sudan OR Sudanese OR Swazi OR Swaziland OR Syria OR Syrian OR East Timor OR East Timorese OR Tonga OR Tongan OR Turkmenistan OR Turkmen OR Tuvalu OR Tuvaluan OR Ukraine OR Ukrainian OR Uzbekistan OR Uzbek OR Vanuatu OR Vietnam OR Vietnamese OR “West Bank” OR Gaza OR Palestinian OR Yemen OR Yemeni OR Yemenite OR Zambia OR Zambian OR Albania OR Albanian OR Algeria OR Algerian OR Argentina OR Argentinian OR Azerbaijan OR Azerbaijani OR Belarus OR Belarusian OR Bosnia OR Bosnian OR Botswana OR Brazil OR Brazilian OR Bulgaria OR Bulgarian OR China OR Chinese OR Colombia OR Colombian OR “Costa Rica” OR “Costa Rican” OR Cuba OR Cuban OR Dominica OR Dominican OR Ecuador OR Ecuadorean OR Gabon OR Gabonese OR Grenada OR Grenadian OR Iran OR Iranian OR Jamaica OR Jamaican OR Jordan OR Jordanian OR Kazakhstan OR Kazakhstani OR	

	Lebanon OR Lebanese OR Libya OR Libyan OR Lithuania OR Lithuanian OR Macedonia OR Macedonian OR Malaysia OR Malaysian OR Maldives OR Maldivian OR Mauritius OR Mauritian OR Mexico OR Mexican OR Montenegro OR Montenegrin OR Namibia OR Namibian OR Palau OR Palauan OR Panama OR Panamanian OR Peru OR Peruvian OR Romania OR Romanian OR Russia OR Russian OR Serbia OR Serbian OR Seychelles OR Seychellois OR "South Africa" OR "South African" OR "Saint Kitts" OR "Saint Lucia" OR "Saint Vincent" OR Surinam OR Suriname OR Surinamer OR Thailand OR Thai OR Tunisia OR Tunisian OR Turkey OR Turkish OR Venezuela OR Venezuala OR Venezuelan OR Venezualan OR Herzegovina OR "Timor Leste" OR "Dominican Republic" OR Grenadines OR "American Samoa" OR "American Samoan" OR "Guinea Bissau" OR "Bissau Guinean")	

EMBASE (through OVID)	1. (developing countries or developing country or medically underserved area or medically underserved areas or LMIC or low income countries or low income country or middle income countries or middle income country or resource poor or low resource or third world country or third world countries or less developed country or less developed countries or least developed country or least developed countries or Africa or Central Asia or Western Asia or Southeastern Asia or Indian Ocean Islands or Central America or South America or Eastern Europe or Transcaucasia or Caribbean Region or Pacific Islands or Afghan or Afghani or Afghanistan or Bangladesh or Bangladeshi or Benin or Beninese or Burkina Faso or Burkinabe or Burundi or Burundian or Cambodia or Cambodian or Central African Republic or Central African or Chad or Chadian or Comoros or Comoran or Congo or Congolese or Eritrea or Eritrean or Ethiopia or Ethiopian or Gambia or Gambian or Guinea or Guinean or Haiti or Haitian or Kenya or Kenyan or Korea or Korean or Kyrgyz or Kyrgyzstan or Liberia or Liberian or Madagascar or Malagasy or Malawi or Malawian or Mali or Malian or Mozambique or Mozambican or Myanmar or Myanmarese or Burmese or Nepal or Nepalese or Niger or Nigerian or Rwanda or Rwandan or Sierra Leone or Sierra Leonean or Somalia or Somalian or Tajikistan or Tajik or Tadzhik or Tanzania or Tanzanian or Togo or Togolese or Uganda or Ugandan or Zimbabwe or Zimbabwean or Angola or Angolan or Armenia or Armenian or Belize or Belizean or Bhutan or Bhutanese or Bolivia or Bolivian or Cameroon or Cameroonian or Cape Verde or Cape Verdean or Cape Verdean or Cote d'Ivoire or Ivory Coast or Djibouti or Egypt or Egyptian or El Salvador or Salvadoran or Fiji or Fijian or Georgia or Georgian or Ghana or Ghanaian or Guatemala or Guatemalan or Guyana or Guyanese or Honduras or Honduran or Indonesia or Indonesian or India or Indian or Iraq or Iraqi or Kiribati or Kosovo or Kosovar or Laos or Lao or Laotian or Lesotho or Marshall Islands or Marshallese or Mauritania or Mauritanian or Micronesia or Micronesian or Moldova or Moldovan or Mongolia or Mongolian or Morocco or Moroccan or Nicaragua or Nicaraguan or Nigeria or Nigerian or Pakistan or Pakistani or Papua New Guinea or Papua New Guinean or Paraguay or Paraguayan or Philippines or Filipino or Samoa or Samoan or Sao Tome or Santomean or Senegal or Senegalese or Solomon Islands or Solomon Islander or Sri Lanka or Sri Lankan or Sudan or Sudanese or Swazi or Swaziland or Syria or Syrian or East Timor or East Timorese or Tonga or Tongan or Turkmenistan or Turkmen or Tuvalu or Tuvaluan or Ukraine or Ukrainian or Uzbekistan or Uzbek or Vanuatu or Vietnam or Vietnamese or West Bank or Gaza or Palestinian or Yemen or		512

	Yemeni or Yemenite or Zambia or Zambian or Albania or Albanian or Algeria or Algerian or Argentina or Argentinian or Azerbaijan or Azerbaijani or Belarus or Belarusian or Bosnia or Bosnian or Botswana or Brazil or Brazilian or Bulgaria or Bulgarian or China or Chinese or Colombia or Colombian or Costa Rica or Costa Rican or Cuba or Cuban or Dominica or Dominican or Ecuador or Ecuadorean or Gabon or Gabonese or Grenada or Grenadian or Iran or Iranian or Jamaica or Jamaican or Jordan or Jordanian or Kazakhstan or Kazakhstani or Lebanon or Lebanese or Libya or Libyan or Lithuania or Lithuanian or Macedonia or Macedonian or Malaysia or Malaysian or Maldives or Maldivian or Mauritius or Mauritian or Mexico or Mexican or Montenegro or Montenegrin or Namibia or Namibian or Palau or Palauan or Panama or Panamanian or Peru or Peruvian or Romania or Romanian or Russia or Russian or Serbia or Serbian or Seychelles or Seychellois or South Africa or South African or Saint Kitts or Saint Lucia or Saint Vincent or Surinam or Suriname or Surinamer or Thailand or Thai or Tunisia or Tunisian or Turkey or Turkish or Venezuela or Venezuala or Venezuelan or Venezualan or Herzegovina or Timor Leste or Dominican Republic or Grenadines or American Samoa or American Samoan or Guinea Bissau or Bissau Guinean).mp. [mp=title, abstract, original title, name of substance word, subject heading word, floating sub-heading word, keyword heading word, organism supplementary concept word, protocol supplementary concept word, rare disease supplementary concept word, unique identifier, synonyms]<br>2. (effect* or impact or odds ratio or relative risk or risk reduction or prevalence or incidence or efficacy or evaluation or epidemiolog*).mp. [mp=title, abstract, original title, name of substance word, subject heading word, floating sub-heading word, keyword heading word, organism supplementary concept word, protocol supplementary concept word, rare disease supplementary concept word, unique identifier, synonyms]<br>3. (season or seasonal or annual or quarterly or community or rural).mp. [mp=title, abstract, original title, name of substance word, subject heading word, floating sub-heading word, keyword heading word, organism supplementary concept word, protocol supplementary concept word, rare disease supplementary concept word, unique identifier, synonyms]<br>4. ((((((((mass test* or mass screen* or focal test* or focal screen* or MSAT or MTAT or FSAT or FTAT or active case finding or active case detection or test) and treat) or screen) and treat) or test-and-treat or screen-and-treat or testing) and treatment) or screening) and treatment).mp. [mp=title, abstract, original title, name of substance word, subject heading word, floating sub-		

	heading word, keyword heading word, organism supplementary concept word, protocol supplementary concept word, rare disease supplementary concept word, unique identifier, synonyms]<br>5. Malaria/<br>6. 1 and 2 and 3 and 4 and 5	

CINALH (through EBSCO host)	malaria<br><br>AND<br><br>(“mass test*” OR “mass screen*” OR “focal test*” OR “focal screen*” OR “MSAT” OR “MTAT” OR “FSAT” OR “FTAT” OR “active case finding” OR “active case detection” OR “test and treat” OR “screen and treat” OR “test-and-treat” OR “screen-and-treat” OR “testing and treatment” OR “screening and treatment”)<br><br>AND<br><br>(season OR seasonal OR annual OR quarterly OR community OR rural)<br><br>AND<br><br>(effect* OR impact OR “odds ratio” OR “relative risk” OR “risk reduction” OR prevalence OR incidence OR efficacy OR evaluation OR epidemiolog* OR “cost-effectiveness” OR “economic evaluation” OR “economic analysis” OR “cost effectiveness”)<br><br>AND<br><br>(“developing countries” OR “developing country” OR “medically underserved area” OR “medically underserved areas” OR LMIC OR “low income countries” OR “low income country” OR “middle income countries” OR “middle income country” OR “resource poor” OR “low resource” OR “third world country” OR “third world countries” OR “less developed country” OR “less developed countries” OR “least developed country” OR “least developed countries” OR Africa OR “Central Asia” OR “Western Asia” OR “Southeastern Asia” OR “Indian Ocean Islands” OR “Central America” OR “South America” OR “Eastern Europe” OR Transcaucasia OR “Caribbean Region” OR “Pacific Islands” OR Afghan OR Afghani OR Afghanistan OR Bangladesh OR Bangladeshi OR Benin OR Beninese OR “Burkina Faso” OR Burkinabe OR Burundi OR Burundian OR Cambodia OR Cambodian OR “Central African Republic” OR “Central African” OR Chad OR Chadian OR Comoros OR Comoran OR Congo OR Congolese OR Eritrea OR Eritrean OR Ethiopia OR Ethiopian OR Gambia OR Gambian OR Guinea OR Guinean OR Haiti OR Haitian OR Kenya OR Kenyan OR Korea OR		30

	Korean OR Kyrgyz OR Kyrgyzstan OR Liberia OR Liberian OR Madagascar OR Malagasy OR Malawi OR Malawian OR Mali OR Malian OR Mozambique OR Mozambican OR Myanmar OR Myanmarese OR Burmese OR Nepal OR Nepalese OR Niger OR Nigerian OR Rwanda OR Rwandan OR “Sierra Leone” OR “Sierra Leonean” OR Somalia OR Somalian OR Tajikistan OR Tajik OR Tadzhik OR Tanzania OR Tanzanian OR Togo OR Togolese OR Uganda OR Ugandan OR Zimbabwe OR Zimbabwean OR Angola OR Angolan OR Armenia OR Armenian OR Belize OR Belizean OR Bhutan OR Bhutanese OR Bolivia OR Bolivian OR Cameroon OR Cameroonian OR “Cape Verde” OR “Cape Verdean” OR “Cape Verdean” OR “Cote d'Ivoire” OR “Ivory Coast” OR Djibouti OR Egypt OR Egyptian OR “El Salvador” OR Salvadoran OR Fiji OR Fijian OR Georgia OR Georgian OR Ghana OR Ghanaian OR Guatemala OR Guatemalan OR Guyana OR Guyanese OR Honduras OR Honduran OR Indonesia OR Indonesian OR India OR Indian OR Iraq OR Iraqi OR Kiribati OR Kosovo OR Kosovar OR Laos OR Lao OR Laotian OR Lesotho OR “Marshall Islands” OR Marshallese OR Mauritania OR Mauritanian OR Micronesia OR Micronesian OR Moldova OR Moldovan OR Mongolia OR Mongolian OR Morocco OR Moroccan OR Nicaragua OR Nicaraguan OR Nigeria OR Nigerian OR Pakistan OR Pakistani OR Papua New Guinea OR Papua New Guinean OR Paraguay OR Paraguayan OR Philippines OR Filipino OR Samoa OR Samoan OR “Sao Tome” OR Santomean OR Senegal OR Senegalese OR “Solomon Islands” OR “Solomon Islander” OR “Sri Lanka” OR “Sri Lankan” OR Sudan OR Sudanese OR Swazi OR Swaziland OR Syria OR Syrian OR East Timor OR East Timorese OR Tonga OR Tongan OR Turkmenistan OR Turkmen OR Tuvalu OR Tuvaluan OR Ukraine OR Ukrainian OR Uzbekistan OR Uzbek OR Vanuatu OR Vietnam OR Vietnamese OR “West Bank” OR Gaza OR Palestinian OR Yemen OR Yemeni OR Yemenite OR Zambia OR Zambian OR Albania OR Albanian OR Algeria OR Algerian OR Argentina OR Argentinian OR Azerbaijan OR Azerbaijani OR Belarus OR Belarusian OR Bosnia OR Bosnian OR Botswana OR Brazil OR Brazilian OR Bulgaria OR Bulgarian OR China OR Chinese OR Colombia OR Colombian OR “Costa Rica” OR “Costa Rican” OR Cuba OR Cuban OR Dominica OR Dominican OR Ecuador OR Ecuadorean OR Gabon OR Gabonese OR Grenada OR Grenadian OR Iran OR Iranian OR Jamaica OR Jamaican OR Jordan OR Jordanian OR Kazakhstan OR Kazakhstani OR Lebanon OR Lebanese OR Libya OR Libyan OR Lithuania OR Lithuanian OR Macedonia OR Macedonian OR Malaysia OR Malaysian OR Maldives OR Maldivian OR Mauritius OR Mauritian OR Mexico OR Mexican OR	

	Montenegro OR Montenegrin OR Namibia OR Namibian OR Palau OR Palauan OR Panama OR Panamanian OR Peru OR Peruvian OR Romania OR Romanian OR Russia OR Russian OR Serbia OR Serbian OR Seychelles OR Seychellois OR "South Africa" OR "South African" OR "Saint Kitts" OR "Saint Lucia" OR "Saint Vincent" OR Surinam OR Suriname OR Surinamer OR Thailand OR Thai OR Tunisia OR Tunisian OR Turkey OR Turkish OR Venezuela OR Venezuala OR Venezuelan OR Venezualan OR Herzegovina OR "Timor Leste" OR "Dominican Republic" OR Grenadines OR "American Samoa" OR "American Samoan" OR "Guinea Bissau" OR "Bissau Guinean")	

	Google scholar	malaria<br><br>AND<br><br>(“mass test*” OR “mass screen*” OR “focal test*” OR “focal screen*” OR “MSAT” OR “MTAT” OR “FSAT” OR “FTAT” OR “active case finding” OR “active case detection” OR “test and treat” OR “screen and treat” OR “test-and-treat” OR “screen-and-treat” OR “testing and treatment” OR “screening and treatment”)<br><br>AND<br><br>(season OR seasonal OR annual OR quarterly OR community OR rural)<br><br>AND<br><br>(effect* OR impact OR “odds ratio” OR “relative risk” OR “risk reduction” OR prevalence OR incidence OR efficacy OR evaluation OR epidemiolog* OR “cost-effectiveness” OR “economic evaluation” OR “economic analysis” OR “cost effectiveness”)<br><br>AND<br><br>(“developing countries” OR “developing country” OR “medically underserved area” OR “medically underserved areas” OR LMIC OR “low income countries” OR “low income country” OR “middle income countries” OR “middle income country” OR “resource poor” OR “low resource” OR “third world country” OR “third world countries” OR “less developed country” OR “less developed countries” OR “least developed country” OR “least developed countries” OR Africa OR “Central Asia” OR “Western Asia” OR “Southeastern Asia” OR “Indian Ocean Islands” OR “Central America” OR “South America” OR “Eastern Europe” OR Transcaucasia OR “Caribbean Region” OR “Pacific Islands” OR Afghan OR Afghani OR Afghanistan OR Bangladesh OR Bangladeshi OR Benin OR Beninese OR “Burkina Faso” OR Burkinabe OR Burundi OR Burundian OR Cambodia OR Cambodian OR “Central African Republic” OR “Central African” OR Chad OR Chadian OR Comoros OR Comoran OR Congo OR Congolese OR Eritrea OR Eritrean OR Ethiopia OR Ethiopian OR Gambia OR Gambian OR Guinea OR Guinean OR Haiti OR Haitian OR Kenya OR Kenyan OR Korea OR	1300

	Korean OR Kyrgyz OR Kyrgyzstan OR Liberia OR Liberian OR Madagascar OR Malagasy OR Malawi OR Malawian OR Mali OR Malian OR Mozambique OR Mozambican OR Myanmar OR Myanmarese OR Burmese OR Nepal OR Nepalese OR Niger OR Nigerian OR Rwanda OR Rwandan OR “Sierra Leone” OR “Sierra Leonean” OR Somalia OR Somalian OR Tajikistan OR Tajik OR Tadzhik OR Tanzania OR Tanzanian OR Togo OR Togolese OR Uganda OR Ugandan OR Zimbabwe OR Zimbabwean OR Angola OR Angolan OR Armenia OR Armenian OR Belize OR Belizean OR Bhutan OR Bhutanese OR Bolivia OR Bolivian OR Cameroon OR Cameroonian OR “Cape Verde” OR “Cape Verdean” OR “Cape Verdean” OR “Cote d'Ivoire” OR “Ivory Coast” OR Djibouti OR Egypt OR Egyptian OR “El Salvador” OR Salvadoran OR Fiji OR Fijian OR Georgia OR Georgian OR Ghana OR Ghanaian OR Guatemala OR Guatemalan OR Guyana OR Guyanese OR Honduras OR Honduran OR Indonesia OR Indonesian OR India OR Indian OR Iraq OR Iraqi OR Kiribati OR Kosovo OR Kosovar OR Laos OR Lao OR Laotian OR Lesotho OR “Marshall Islands” OR Marshallese OR Mauritania OR Mauritanian OR Micronesia OR Micronesian OR Moldova OR Moldovan OR Mongolia OR Mongolian OR Morocco OR Moroccan OR Nicaragua OR Nicaraguan OR Nigeria OR Nigerian OR Pakistan OR Pakistani OR Papua New Guinea OR Papua New Guinean OR Paraguay OR Paraguayan OR Philippines OR Filipino OR Samoa OR Samoan OR “Sao Tome” OR Santomean OR Senegal OR Senegalese OR “Solomon Islands” OR “Solomon Islander” OR “Sri Lanka” OR “Sri Lankan” OR Sudan OR Sudanese OR Swazi OR Swaziland OR Syria OR Syrian OR East Timor OR East Timorese OR Tonga OR Tongan OR Turkmenistan OR Turkmen OR Tuvalu OR Tuvaluan OR Ukraine OR Ukrainian OR Uzbekistan OR Uzbek OR Vanuatu OR Vietnam OR Vietnamese OR “West Bank” OR Gaza OR Palestinian OR Yemen OR Yemeni OR Yemenite OR Zambia OR Zambian OR Albania OR Albanian OR Algeria OR Algerian OR Argentina OR Argentinian OR Azerbaijan OR Azerbaijani OR Belarus OR Belarusian OR Bosnia OR Bosnian OR Botswana OR Brazil OR Brazilian OR Bulgaria OR Bulgarian OR China OR Chinese OR Colombia OR Colombian OR “Costa Rica” OR “Costa Rican” OR Cuba OR Cuban OR Dominica OR Dominican OR Ecuador OR Ecuadorean OR Gabon OR Gabonese OR Grenada OR Grenadian OR Iran OR Iranian OR Jamaica OR Jamaican OR Jordan OR Jordanian OR Kazakhstan OR Kazakhstani OR Lebanon OR Lebanese OR Libya OR Libyan OR Lithuania OR Lithuanian OR Macedonia OR Macedonian OR Malaysia OR Malaysian OR Maldives OR Maldivian OR Mauritius OR Mauritian OR Mexico OR Mexican OR	

	Montenegro OR Montenegrin OR Namibia OR Namibian OR Palau OR Palauan OR Panama OR Panamanian OR Peru OR Peruvian OR Romania OR Romanian OR Russia OR Russian OR Serbia OR Serbian OR Seychelles OR Seychellois OR “South Africa” OR “South African” OR “Saint Kitts” OR “Saint Lucia” OR “Saint Vincent” OR Surinam OR Suriname OR Surinamer OR Thailand OR Thai OR Tunisia OR Tunisian OR Turkey OR Turkish OR Venezuela OR Venezuala OR Venezuelan OR Venezualan OR Herzegovina OR “Timor Leste” OR “Dominican Republic” OR Grenadines OR “American Samoa” OR “American Samoan” OR “Guinea Bissau” OR “Bissau Guinean”)	

Web of science	malaria<br><br>AND<br><br>(mass test* OR mass screen* OR focal test* OR focal screen* OR MSAT OR MTAT OR FSAT OR FTAT OR active case finding OR active case detection OR test and treat OR screen and treat OR test-and-treat OR screen-and-treat OR testing and treatment OR screening and treatment)<br><br>AND<br><br>(season OR seasonal OR annual OR quarterly OR community OR rural)<br><br>AND<br><br>(effect* OR impact OR odds ratio OR relative risk OR risk reduction OR prevalence OR incidence OR efficacy OR evaluation OR epidemiolog*)<br><br>AND<br><br>(developing countries OR developing country OR medically underserved area OR medically underserved areas OR LMIC OR low income countries OR low income country OR middle income countries OR middle income country OR resource poor OR low resource OR third world country OR third world countries OR less developed country OR less developed countries OR least developed country OR least developed countries OR Africa OR Central Asia OR Western Asia OR Southeastern Asia OR Indian Ocean Islands OR Central America OR South America OR Eastern Europe OR Transcaucasia OR Caribbean Region OR Pacific Islands OR Afghan OR Afghani OR Afghanistan OR Bangladesh OR Bangladeshi OR Benin OR Beninese OR Burkina Faso OR Burkinabe OR Burundi OR Burundian OR Cambodia OR Cambodian OR Central African Republic OR Central African OR Chad OR Chadian OR Comoros OR Comoran OR Congo OR Congolese OR Eritrea OR Eritrean OR Ethiopia OR Ethiopian OR Gambia OR Gambian OR Guinea OR Guinean OR Haiti OR Haitian OR Kenya OR Kenyan OR Korea OR Korean OR Kyrgyz OR Kyrgyzstan OR Liberia OR Liberian OR Madagascar OR Malagasy OR Malawi OR Malawian OR Mali OR Malian OR Mozambique OR Mozambican OR Myanmar OR Myanmarese OR Burmese OR Nepal OR Nepalese OR Niger OR Nigerian OR Rwanda OR	21

	Rwandan OR Sierra Leone OR Sierra Leonean OR Somalia OR Somalian OR Tajikistan OR Tajik OR Tadzhik OR Tanzania OR Tanzanian OR Togo OR Togolese OR Uganda OR Ugandan OR Zimbabwe OR Zimbabwean OR Angola OR Angolan OR Armenia OR Armenian OR Belize OR Belizean OR Bhutan OR Bhutanese OR Bolivia OR Bolivian OR Cameroon OR Cameroonian OR Cape Verde OR Cape Verdean OR Cape Verdean OR Cote d'Ivoire OR Ivory Coast OR Djibouti OR Egypt OR Egyptian OR El Salvador OR Salvadoran OR Fiji OR Fijian OR Georgia OR Georgian OR Ghana OR Ghanaian OR Guatemala OR Guatemalan OR Guyana OR Guyanese OR Honduras OR Honduran OR Indonesia OR Indonesian OR India OR Indian OR Iraq OR Iraqi OR Kiribati OR Kosovo OR Kosovar OR Laos OR Lao OR Laotian OR Lesotho OR Marshall Islands OR Marshallese OR Mauritania OR Mauritanian OR Micronesia OR Micronesian OR Moldova OR Moldovan OR Mongolia OR Mongolian OR Morocco OR Moroccan OR Nicaragua OR Nicaraguan OR Nigeria OR Nigerian OR Pakistan OR Pakistani OR Papua New Guinea OR Papua New Guinean OR Paraguay OR Paraguayan OR Philippines OR Filipino OR Samoa OR Samoan OR Sao Tome OR Santomean OR Senegal OR Senegalese OR Solomon Islands OR Solomon Islander OR Sri Lanka OR Sri Lankan OR Sudan OR Sudanese OR Swazi OR Swaziland OR Syria OR Syrian OR East Timor OR East Timorese OR Tonga OR Tongan OR Turkmenistan OR Turkmen OR Tuvalu OR Tuvaluan OR Ukraine OR Ukrainian OR Uzbekistan OR Uzbek OR Vanuatu OR Vietnam OR Vietnamese OR West Bank OR Gaza OR Palestinian OR Yemen OR Yemeni OR Yemenite OR Zambia OR Zambian OR Albania OR Albanian OR Algeria OR Algerian OR Argentina OR Argentinian OR Azerbaijan OR Azerbaijani OR Belarus OR Belarusian OR Bosnia OR Bosnian OR Botswana OR Brazil OR Brazilian OR Bulgaria OR Bulgarian OR China OR Chinese OR Colombia OR Colombian OR Costa Rica OR Costa Rican OR Cuba OR Cuban OR Dominica OR Dominican OR Ecuador OR Ecuadorean OR Gabon OR Gabonese OR Grenada OR Grenadian OR Iran OR Iranian OR Jamaica OR Jamaican OR Jordan OR Jordanian OR Kazakhstan OR Kazakhstani OR Lebanon OR Lebanese OR Libya OR Libyan OR Lithuania OR Lithuanian OR Macedonia OR Macedonian OR Malaysia OR Malaysian OR Maldives OR Maldivian OR Mauritius OR Mauritian OR Mexico OR Mexican OR Montenegro OR Montenegrin OR Namibia OR Namibian OR Palau OR Palauan OR Panama OR Panamanian OR Peru OR Peruvian OR Romania OR Romanian OR Russia OR Russian OR Serbia OR Serbian OR Seychelles OR Seychellois OR South Africa OR South African OR Saint Kitts OR Saint Lucia OR Saint	

	Vincent OR Surinam OR Suriname OR Surinamer OR Thailand OR Thai OR Tunisia OR Tunisian OR Turkey OR Turkish OR Venezuela OR Venezuala OR Venezuelan OR Venezualan OR Herzegovina OR Timor Leste OR Dominican Republic OR Grenadines OR American Samoa OR American Samoan OR Guinea Bissau OR Bissau Guinean)	

Global Health (through OVID)	1. (developing countries or developing country or medically underserved area or medically underserved areas or LMIC or low income countries or low income country or middle income countries or middle income country or resource poor or low resource or third world country or third world countries or less developed country or less developed countries or least developed country or least developed countries or Africa or Central Asia or Western Asia or Southeastern Asia or Indian Ocean Islands or Central America or South America or Eastern Europe or Transcaucasia or Caribbean Region or Pacific Islands or Afghan or Afghani or Afghanistan or Bangladesh or Bangladeshi or Benin or Beninese or Burkina Faso or Burkinabe or Burundi or Burundian or Cambodia or Cambodian or Central African Republic or Central African or Chad or Chadian or Comoros or Comoran or Congo or Congolese or Eritrea or Eritrean or Ethiopia or Ethiopian or Gambia or Gambian or Guinea or Guinean or Haiti or Haitian or Kenya or Kenyan or Korea or Korean or Kyrgyz or Kyrgyzstan or Liberia or Liberian or Madagascar or Malagasy or Malawi or Malawian or Mali or Malian or Mozambique or Mozambican or Myanmar or Myanmarese or Burmese or Nepal or Nepalese or Niger or Nigerian or Rwanda or Rwandan or Sierra Leone or Sierra Leonean or Somalia or Somalian or Tajikistan or Tajik or Tadzhik or Tanzania or Tanzanian or Togo or Togolese or Uganda or Ugandan or Zimbabwe or Zimbabwean or Angola or Angolan or Armenia or Armenian or Belize or Belizean or Bhutan or Bhutanese or Bolivia or Bolivian or Cameroon or Cameroonian or Cape Verde or Cape Verdean or Cape Verdean or Cote d'Ivoire or Ivory Coast or Djibouti or Egypt or Egyptian or El Salvador or Salvadoran or Fiji or Fijian or Georgia or Georgian or Ghana or Ghanaian or Guatemala or Guatemalan or Guyana or Guyanese or Honduras or Honduran or Indonesia or Indonesian or India or Indian or Iraq or Iraqi or Kiribati or Kosovo or Kosovar or Laos or Lao or Laotian or Lesotho or Marshall Islands or Marshallese or Mauritania or Mauritanian or Micronesia or Micronesian or Moldova or Moldovan or Mongolia or Mongolian or Morocco or Moroccan or Nicaragua or Nicaraguan or Nigeria or Nigerian or Pakistan or Pakistani or Papua New Guinea or Papua New Guinean or Paraguay or Paraguayan or Philippines or Filipino or Samoa or Samoan or Sao Tome or Santomean or Senegal or Senegalese or Solomon Islands or Solomon Islander or Sri Lanka or Sri Lankan or Sudan or Sudanese or Swazi or Swaziland or Syria or Syrian or East Timor or East Timorese or Tonga or Tongan or Turkmenistan or Turkmen or Tuvalu or Tuvaluan or Ukraine or Ukrainian or Uzbekistan or Uzbek or Vanuatu or Vietnam or Vietnamese or West Bank or Gaza or Palestinian or Yemen or	297

	Yemeni or Yemenite or Zambia or Zambian or Albania or Albanian or Algeria or Algerian or Argentina or Argentinian or Azerbaijan or Azerbaijani or Belarus or Belarusian or Bosnia or Bosnian or Botswana or Brazil or Brazilian or Bulgaria or Bulgarian or China or Chinese or Colombia or Colombian or Costa Rica or Costa Rican or Cuba or Cuban or Dominica or Dominican or Ecuador or Ecuadorean or Gabon or Gabonese or Grenada or Grenadian or Iran or Iranian or Jamaica or Jamaican or Jordan or Jordanian or Kazakhstan or Kazakhstani or Lebanon or Lebanese or Libya or Libyan or Lithuania or Lithuanian or Macedonia or Macedonian or Malaysia or Malaysian or Maldives or Maldivian or Mauritius or Mauritian or Mexico or Mexican or Montenegro or Montenegrin or Namibia or Namibian or Palau or Palauan or Panama or Panamanian or Peru or Peruvian or Romania or Romanian or Russia or Russian or Serbia or Serbian or Seychelles or Seychellois or South Africa or South African or Saint Kitts or Saint Lucia or Saint Vincent or Surinam or Suriname or Surinamer or Thailand or Thai or Tunisia or Tunisian or Turkey or Turkish or Venezuela or Venezuala or Venezuelan or Venezualan or Herzegovina or Timor Leste or Dominican Republic or Grenadines or American Samoa or American Samoan or Guinea Bissau or Bissau Guinean).mp. [mp=title, abstract, original title, name of substance word, subject heading word, floating sub-heading word, keyword heading word, organism supplementary concept word, protocol supplementary concept word, rare disease supplementary concept word, unique identifier, synonyms]<br>2. (effect* or impact or odds ratio or relative risk or risk reduction or prevalence or incidence or efficacy or evaluation or epidemiolog*).mp. [mp=title, abstract, original title, name of substance word, subject heading word, floating sub-heading word, keyword heading word, organism supplementary concept word, protocol supplementary concept word, rare disease supplementary concept word, unique identifier, synonyms]<br>3. (season or seasonal or annual or quarterly or community or rural).mp. [mp=title, abstract, original title, name of substance word, subject heading word, floating sub-heading word, keyword heading word, organism supplementary concept word, protocol supplementary concept word, rare disease supplementary concept word, unique identifier, synonyms]<br>4. ((((((((mass test* or mass screen* or focal test* or focal screen* or MSAT or MTAT or FSAT or FTAT or active case finding or active case detection or test) and treat) or screen) and treat) or test-and-treat or screen-and-treat or testing) and treatment) or screening) and treatment).mp. [mp=title, abstract, original title, name of substance word, subject heading word, floating sub-	

	heading word, keyword heading word, organism supplementary concept word, protocol supplementary concept word, rare disease supplementary concept word, unique identifier, synonyms]<br>5. Malaria/<br>6. 1 and 2 and 3 and 4 and 5	

3. Data used in meta-analyses

Table S3.1. Data used for the pooled incidence risk ratio (IRR) of the incidence

Study	Measured outcome	ITT or PP (if specified)	Coefficient	95% CI - Min	95% CI - Max
Sutanto et al, 2018*	Hazard ratio of malaria incidence in school children (PCR)	NA	0.98	0.63	1.51
Larsen et al, 2015	Monthly outpatient malaria incidence (suspected + confirmed, IRR in DiD)	ITT	0.65	0.54	0.78
Desai et al, 2020	Pooled annual cumulative incidence of all microscopy-confirmed malaria incidence (IRR)	ITT	0.95	0.87	1.04
Conner et al, 2020	Intervention effect on average incidence malaria cases per week based on community health facility data + cases detected during MTAT and PECADOM (IRR measured by DiD using negative binomial regression)	ITT	0.76	0.56	1.04

*Hazard ratio (HR) instead of incidence rate ratio (IRR) used

Table S3.2. Data used for the pooled relative risk (RR) of the prevalence

Study	Measured outcome	ITT or PP (if specified)	Coefficient	95% CI - Min	95% CI - Max
Tiono et al (a), 2013,	Prevalence of asymptomatic carrier - Campaign 4/Day1 (mean %)	PP	0.91	0.53	1.66
Samuels et al, 2020	Adjusted ratio of prevalence ratio (aRPR) of malaria by microscopy at year 2 - all ages	ITT	0.92	0.76	1.10
Halliday et al, 2014	Adjusted risk ratio (RR) for *P.falciparum* prevalence	ITT	1.53	0.89	2.62

Table S3.3. Data used for the pooled odds rato (OR) of the prevalence

Study	Measured outcome	ITT or PP (if specified)	Coefficient	95% CI - Min	95% CI - Max
Larsen et al, 2015	malaria parasite prevalence in children aged 1- 59 months (high season) - adjusted OR	PP	0.55	0.29	1.06
Sutanto et al, 2018	*P.falciparum* prevalence by PCR post intervention - cluster level	NA	0.25	0.21	0.31

4. Sub-group analysis on pregnant women

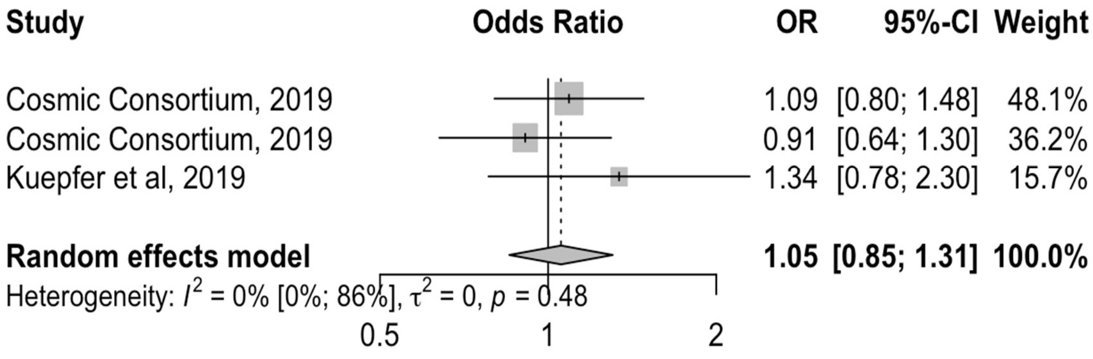

Figure S4.1. Pooled estimate of the odds ratio (OR) of placental malaria infection

Table S4.1. Pooled estimate of the odds ratio (OR) of placental malaria infection

Study	Measured outcome	ITT or PP (if specified)	Coefficient	95% CI - Min	95% CI - Max
Cosmic Consortium, 2019	Placental malaria incidence in Burkina Faso and Gambia (any infection)	PP	1.09	0.80	1.48
Cosmic Consortium, 2019	Adjusted OR of placental malaria (any infection) - Burkina Faso	PP	0.91	0.64	1.30
Kuepfer et al, 2019	Any placental malaria upon delivery (for those who delivered at the institution only)	ITT	1.34	0.78	2.29

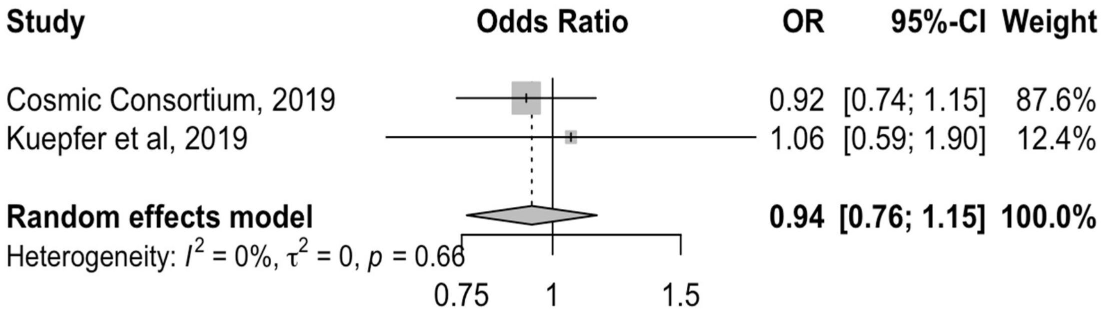

Figure S4.2. Pooled estimate of the odds ratio (OR) of peripheral infection

Table S4.2. Pooled estimate of the odds ratio (OR) of peripheral infection

Study	Measured outcome	ITT or PP (if specified)	Coefficient	95% CI - Min	95% CI - Max
Cosmic Consortium, 2019	Adjusted OR for maternal peripheral infection (measured by PCR)	PP	0.92	0.74	1.15
Kuepfer et al, 2019	Maternal peripheral parasitemia (PCR)	ITT	1.06	0.59	1.90

5. **Funnel plots for meta-analysis**

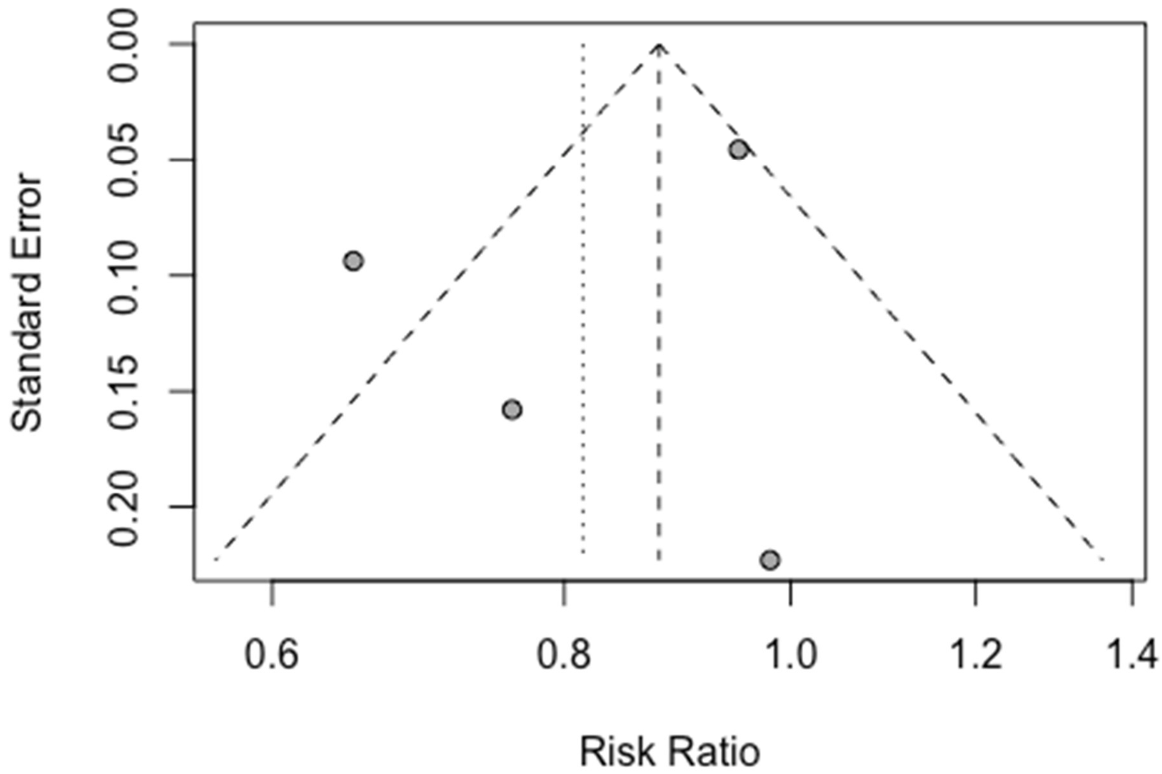

Figure S5.1. Funnel plot for pooled risk ratio of incidence (corresponding to Figure 2-A) using random-effect meta-analysis methods

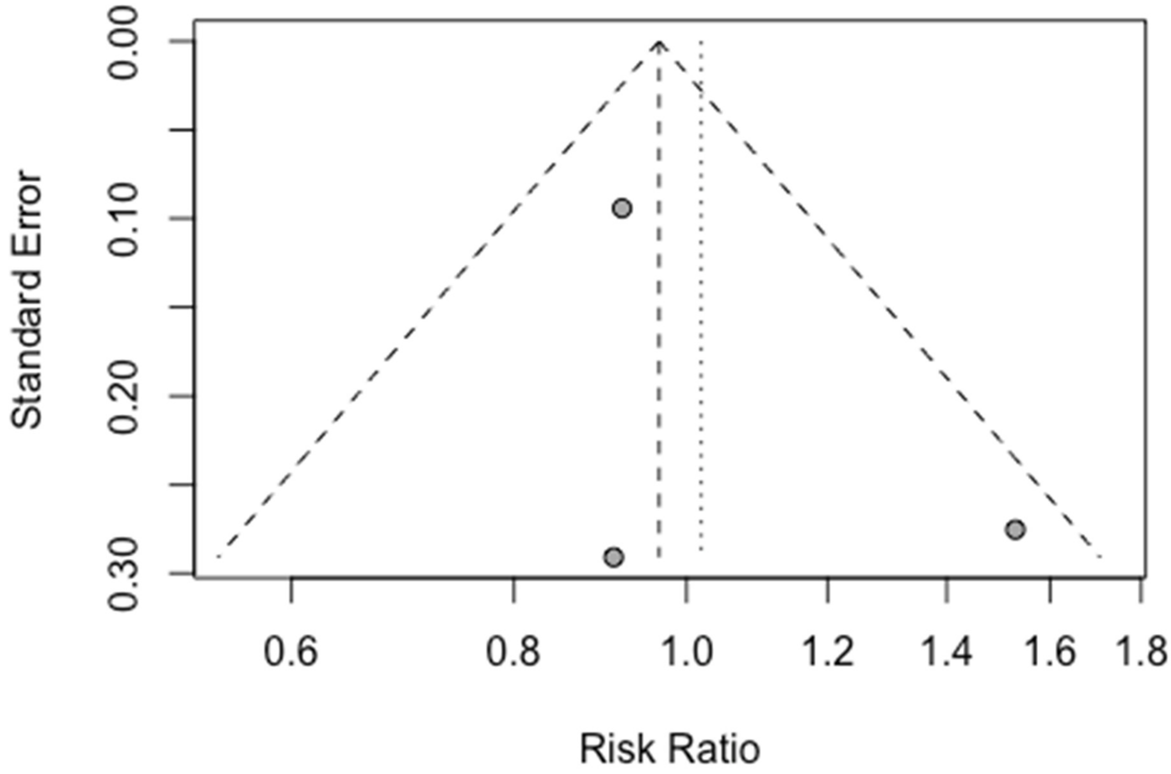

Figure S5.2. Funnel plot for pooled risk ratio of prevalence (corresponding to Figure 2-B) using random-effect meta-analysis methods

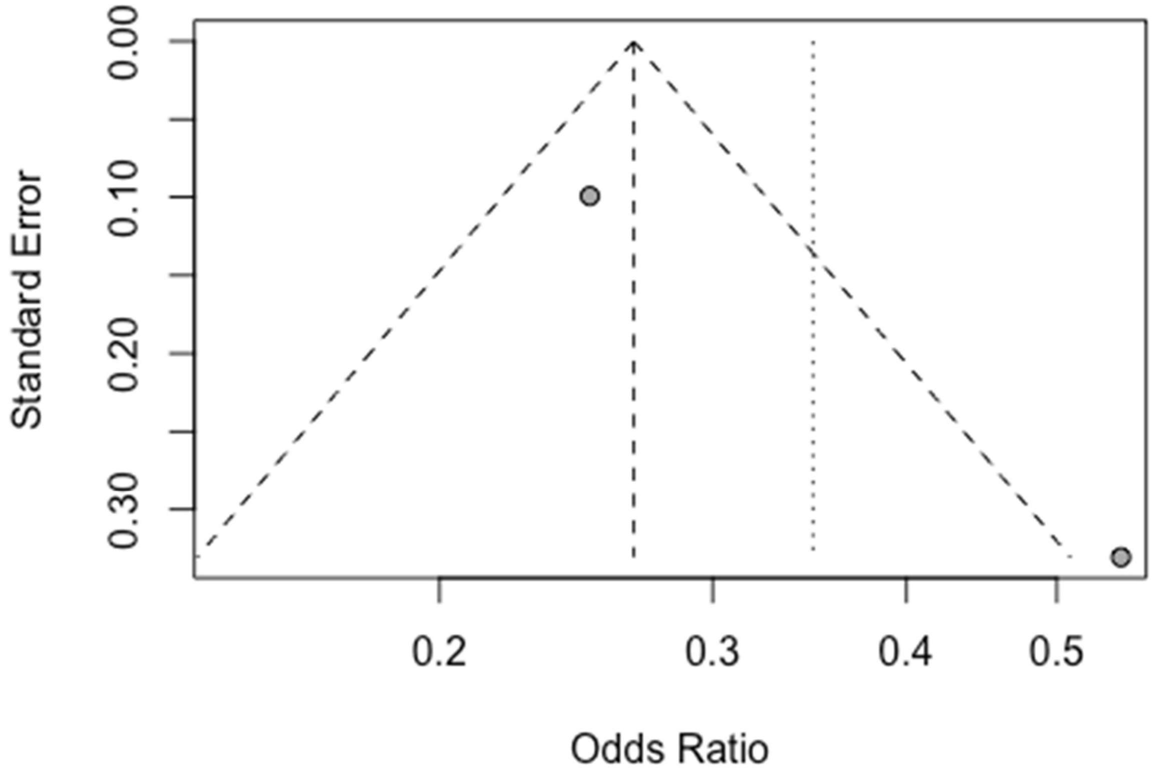

Figure S5.1. Funnel plot for pooled odds ratio of prevalence (corresponding to Figure 2-C) using random-effect meta-analysis methods
